# Indoor Residual Spraying Delivery Models to Prevent Malaria: Comparison of Community- and District-Based Approaches in Ethiopia

**DOI:** 10.9745/GHSP-D-16-00165

**Published:** 2016-12-23

**Authors:** Benjamin Johns, Yemane Yeebiyo Yihdego, Lena Kolyada, Dereje Dengela, Sheleme Chibsa, Gunawardena Dissanayake, Kristen George, Hiwot Solomon Taffese, Bradford Lucas

**Affiliations:** a Abt Associates, Inc., The President's Malaria Initiative Africa Indoor Residual Spraying Project, Bethesda, MD, USA.; b Abt Associates, Inc., The President's Malaria Initiative Africa Indoor Residual Spraying Project, Addis Ababa, Ethiopia.; c U.S. President's Malaria Initiative, U.S. Agency for International Development, Addis Ababa, Ethiopia.; d U.S. President's Malaria Initiative, U.S. Agency for International Development, Bureau for Global Health, Office of Health, Infectious Disease & Nutrition, Arlington, VA, USA.; e National Malaria Control Program, Federal Ministry of Health, Addis Ababa, Ethiopia.

## Abstract

**Background:**

Indoor residual spraying (IRS) for malaria prevention has traditionally been implemented in Ethiopia by the district health office with technical and operational inputs from regional, zonal, and central health offices. The United States President's Malaria Initiative (PMI) in collaboration with the Government of Ethiopia tested the effectiveness and efficiency of integrating IRS into the government-funded community-based rural health services program.

**Methods:**

Between 2012 and 2014, PMI conducted a mixed-methods study in 11 districts of Oromia region to compare district-based IRS (DB IRS) and community-based IRS (CB IRS) models. In the DB IRS model, each district included 2 centrally located operational sites where spray teams camped during the IRS campaign and from which they traveled to the villages to conduct spraying. In the CB IRS model, spray team members were hired from the communities in which they operated, thus eliminating the need for transport and camping facilities. The study team evaluated spray coverage, the quality of spraying, compliance with environmental and safety standards, and cost and performance efficiency.

**Results:**

The average number of eligible structures found and sprayed in the CB IRS districts increased by 19.6% and 20.3%, respectively, between 2012 (before CB IRS) and 2013 (during CB IRS). Between 2013 and 2014, the numbers increased by about 14%. In contrast, in the DB IRS districts the number of eligible structures found increased by only 8.1% between 2012 and 2013 and by 0.4% between 2013 and 2014. The quality of CB IRS operations was good and comparable to that in the DB IRS model, according to wall bioassay tests. Some compliance issues in the first year of CB IRS implementation were corrected in the second year, bringing compliance up to the level of the DB IRS model. The CB IRS model had, on average, higher amortized costs per district than the DB IRS model but lower unit costs per structure sprayed and per person protected because the community-based model found and sprayed more structures.

**Conclusion:**

Established community-based service delivery systems can be adapted to include a seasonal IRS campaign alongside the community-based health workers' routine activities to improve performance efficiency. Further modifications of the community-based IRS model may reduce the total cost of the intervention and increase its financial sustainability.

## Abstract (precis)

Integrating indoor residual spraying into the institutionalized community-based health system in 5 districts was more efficient than the district-based model and did not compromise quality or compliance with environmental standards.

## BACKGROUND

Indoor residual spraying (IRS) is one of the primary methods, along with long-lasting insecticide-treated nets, used to control and reduce the burden of malaria.^1^^,^^2^ IRS involves spraying insecticide on the walls, ceilings, and other indoor resting places of mosquitoes that transmit malaria. In most cases, eligible structures targeted for spraying are the sleeping and living rooms of a household. On average, an effective IRS campaign, regardless of the size of the operation, requires 30–35 days and takes place once or twice a year based on the malaria transmission season and the duration of effective action of the insecticide used in a country. It is a complex operation that often involves hundreds of personnel, including seasonal workers and full-time government employees.

Indoor residual spraying (IRS) campaigns to prevent malaria are often complex operations involving hundreds of personnel.

In recent years, use of IRS has expanded in many African countries, primarily through support from the United States President's Malaria Initiative (PMI) and the Global Fund to Fight AIDS, Tuberculosis and Malaria.^3^^,^^4^ The population at risk for malaria protected by IRS increased from about 5% in 2005 to about 37% in 2013, according to the *World Malaria Report 2014*.^5^

In Ethiopia, IRS has been continuously implemented since it was introduced in the 1950s.^6^ PMI started supporting IRS in Ethiopia in 2008. Survey data show that in 2011, through PMI's and the national government's spray program, IRS protected about 17% of the 50 million people at risk of malaria.^7^

Growing vector resistance to DDT (dichlorodiphenyltrichlorethane) and pyrethroid insecticides in Ethiopia has resulted in the need to switch to more expensive insecticide classes (carbamates and organophosphates). This has stressed limited budgets and may result in a decline of coverage.^8^^–^^9^ Further, international funding for malaria control may have plateaued,^10^ and therefore countries may have to deploy (already limited) domestic resources to expand IRS protection.

In the face of these fiscal pressures, improving the efficiency of delivering IRS is a means of increasing coverage without increasing the resources needed. A recent review suggested that community-based malaria interventions, including bed net distribution, IRS, intermittent preventive therapy, and education, may be more efficient than routine or facility-based modes of implementation.^11^ However, the review found only one study that assessed IRS—a study in China evaluating a program that implemented IRS while delivering insecticide-treated nets at the same time.^12^ Preliminary evidence from Tanzania suggests that community-based approaches for IRS show promise both in terms of coverage and costs, but a full evaluation has not yet been completed.^13^ The purpose of this study is to compare a community-based approach to IRS used in Ethiopia with the traditional district-based approach.

Community-based IRS approaches may be more efficient than routine models, but they have not been evaluated fully.

### Ethiopia's Community-Based Health Extension Program

Over the last several decades, there has been an expansion of community-based programs employing multiple interventions to achieve population-level impact on disease prevention and health promotion. Community participation and ownership are vital for generating community support and capacity for engaging in prevention activities.^14^^–^^17^

Ethiopia has been implementing community-based health services through its Health Extension Program (HEP) since 2005. The HEP is a government-funded health service delivery program that aims for universal coverage of primary health care and equitable access to health services. The program prioritizes prevention and control of communicable diseases and has shown remarkable achievements in the reduction of maternal and child mortality and in the number of communicable disease cases.^18^^–^^22^

As a preventive program, the HEP focuses on 4 areas of care provided at the community level: disease prevention and control; family health, hygiene, and environmental sanitation; health education; and communication. Key health areas under the HEP's aegis are: HIV/AIDS, tuberculosis, malaria, and first aid. To deliver these services, the HEP is expanding its health infrastructure and developing a cadre of paid health extension workers (HEWs) who provide the services to the communities. The HEWs are typically young women with a high school diploma, whom the Government of Ethiopia employs after they complete a 1-year HEP training course. The HEP deploys 2 HEWs in every village of about 5,000 residents.^23^^–^^26^ Currently, there are about 34,000 HEWs in 15,000 rural communities in Ethiopia.

### The District- and Community-Based IRS Models in Ethiopia

In Ethiopia, district health offices have traditionally planned and implemented IRS with guidance from regional and central health offices. Similarly, PMI-supported areas use a district-based IRS (DB IRS) model—that is, district health offices are in charge of planning and implementing IRS with technical and logistical support from PMI partners. Each district, on average, includes 2 centrally located operational sites, where the spray teams stay for the duration of the IRS campaign. Camping accommodations include tents, mattresses, and other items. The spray team comprises a team leader, up to 4 squad leaders and porters, and 16–20 spray operators (SOPs). The number of spray teams depends on the number of structures to be sprayed in the district. SOPs require vehicles on a daily basis to travel from the operational sites to villages to conduct spraying.

Ethiopia has started to shift IRS implementation to the community level by incorporating it into the Health Extension Program.

The Government of Ethiopia has started to shift implementation of IRS to the community level by incorporating the planning and execution of the operation into the HEP. The main reasons for this shift are to: (1) increase spray coverage; (2) increase community participation and ownership; and (3) reduce costs and make IRS more sustainable. Where IRS is integrated into the HEP, HEWs fulfill the role and responsibilities of the squad leaders. They manage all IRS processes at the village level, which usually last for 1–2 months a year. The main duties that HEWs assume are to lead the squad in spraying the community and ensure SOPs follow safety procedures and clean their equipment per standard requirements. In consultations with village leadership, the HEWs select 5 SOPs to train and to conduct the spraying. HEWs also assume responsibility for mobilizing the community; managing store rooms and insecticide stocks, washers, and operators; and overseeing the data collection and reporting processes for their squad. Both HEWs in each village are trained on IRS techniques and management. However, to avoid any disruption with routine HEP activities, only 1 HEW per village leads the spray squad during the spray operation while the other HEW carries out routine HEP duties.

**Figure:**
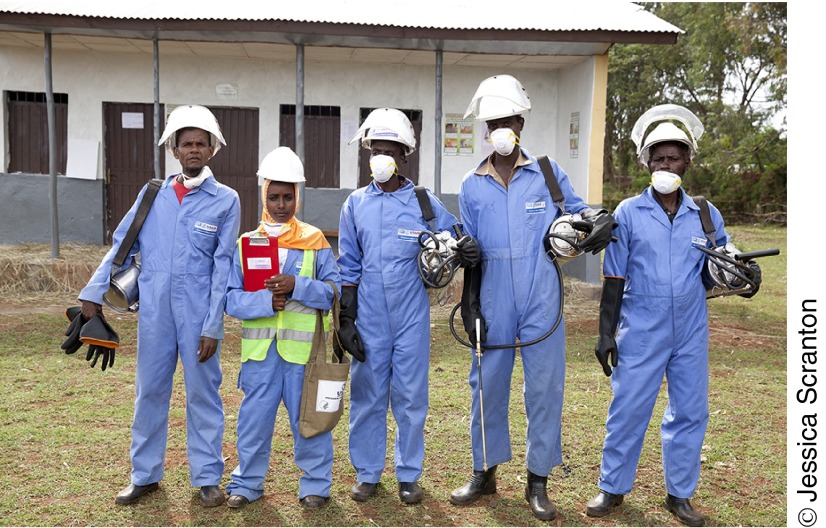
In Ethiopia's community-based indoor residual spraying model, a health extension worker (second from the left) serves as the squad leader for a team of spray operators.

Because in this community-based IRS (CB IRS) implementation model SOPs and squad leaders are hired from the communities in which they operate, there is no need for transport or for camping facilities as required in DB IRS. However, the district health office continues to plan IRS activities, allocate re-sources, and supervise spraying operations and the members of the spray squads. The CB IRS model has never been systematically evaluated.

In the community-based IRS model, spray operators and squad leaders are hired from the communities in which they operate, eliminating the need for transport and camping facilities as required in the district-based model.

### Purpose of the Study

The PMI Africa IRS (PMI AIRS) Project assists Ethiopia with IRS planning, operations, environmental compliance, vector monitoring, and logistics. As part of these efforts, the project conducted this study to compare DB IRS and CB IRS approaches. By comparing the performance of the districts under the new model of CB IRS with the traditional model of DB IRS, the study aimed to assess if using the HEP platform could reduce costs, increase community acceptance, and make operations more sustainable while maintaining high quality and compliance with safety and environmental standards.

## METHODOLOGY

### Selection of Districts

**FIGURE 1.**
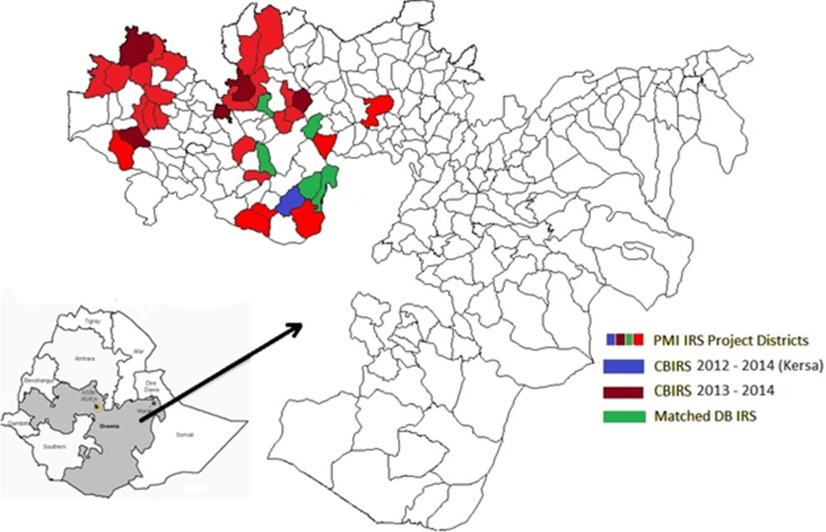
Location of CB IRS and Matched DB IRS Districts, Ethiopia, 2012–2014 Abbreviations: CB IRS, community-based indoor residual spraying; DB IRS, district-based indoor residual spraying; IRS, indoor residual spraying; PMI, United States President's Malaria Initiative.

In 2012, the PMI AIRS Project collaborated with the Government of Ethiopia to pilot the CB IRS model in Kersa district, located in Jimma Zone, Oromia Region, one of 36 PMI-supported districts (Figure 1). PMI and government officials deemed that the CB IRS pilot showed proof of concept that CB IRS could be implemented feasibly, and thus in 2013 they selected 5 more districts to shift from DB IRS to CB IRS to further test the CB IRS model: Bako Tibe, Chewaka, Hawa Galan, Manasibu, and Sasiga. The selection of the 5 additional districts to start CB IRS was primarily based on the districts with the highest number of structures found by the project in 2012, which was used as a target for the following year's spray operations. These 5 CB IRS districts were matched with 5 DB IRS districts that had a comparable number of structures found in 2012: Borecha, Dano, Sekoru, Tiro Afeta, Wayu Tuka, (Supplementary Material 1). Proximity and accessibility of the districts were also considerations in matching the 2 sets of districts. Matching was done before the start of CB IRS in the 5 selected districts.

The project collected data on spray coverage (eligible structures found and sprayed), the quality of spraying, safety and environmental compliance, and cost. The sampling for each data collection method is provided in Table 1.

**TABLE 1.** Data Collection Sampling and Methods, Community- vs. District-Based IRS, Ethiopia, 2012–2014

Purpose of Evaluation	Data Collection Method	Districts		Sites		Process/Outcome Indicators
		CB IRS	DB IRS	CB IRS	DB IRS	
Spray coverage	Spray Operator Form, data collected daily	5	5	5	5	Number of eligible structures found; number of eligible structures sprayed; spray coverage rate; total population protected
Quality of spraying	Wall bioassays, 2013/2014	6/2	2/2	6/2	2/2	Mortality rate of mosquitoes
Environmental compliance	13-item checklist	6	30	30	30	Compliance with best management practices
Cost analysis	Before-after analysis in CB IRS districts (2012 vs. 2013 and 2014) and comparison of CB IRS with matched DB IRS districts (2013 and 2014)	5	5	—	—	Total costs; cost per structure sprayed; cost per person protected

### Selection and Training of HEWs and Spray Teams

Two HEWs from each village in all CB IRS districts received 6 days of training on IRS tech-niques and management. In general, all IRS actors must receive training every year before a spray campaign. Because many of the actors continue with the spray campaign year after year, the annual trainings often serve as refresher trainings for experienced implementers including HEWs. At the same time, the training program has a special focus on new actors, who are usually SOPs and squad leaders.

The PMI AIRS Project tested the length of the training in a number of countries over the years and confirmed that a 6-day curriculum is sufficient to ensure comprehension of IRS standards and to offer sufficient practice time of the spraying techniques. In Ethiopia, this approach has been in use for several decades, and revisions to the curriculum are made when the World Health Organization (WHO) or the Ministry of Health issues additional guidance.

District malaria teams organized and facilitated the training for HEWs with technical and logistical support from the PMI AIRS Project. Following the training at the district level, HEWs returned to their respective villages and, in collaboration with community leaders, selected 5 members of the community to be trained as SOPs. Literacy, acceptance by the community, physical fitness, and previous experience as an SOP were criteria in the selection process. Then, in each village, 2 HEWs trained the selected SOPs on techniques and related aspects of IRS. District health offices provided minimal supervision to the training.

### Spray Coverage

In both IRS delivery models, squad leaders (HEWs in the CB IRS model) collected data on number of structures sprayed on a daily basis using the Daily Spray Operator Form. At the end of the day, seasonal data entry clerks located at the district data centers entered data into an electronic database. The project used 3 data quality assurance tools (the Error Eliminator Form, Data Collection Verification Form, and Data Entry Verification Form) to ensure proper supervision of data collection and data entry. Additionally, the PMI AIRS project used the AIRS Access Database Cleaning/Reporting Tool, which is linked to the PMI AIRS database backend (i.e., the spray data) and has 2 functions: generating district-level reports and data cleaning. The district-level reports provide spray progress to date, per day, per week, per squad, per administrative level (district, village), per spray operator, etc., with the refined data.

### Quality Control

To compare quality of spraying between the 2 models, the study team used a test method (WHO wall bioassays) to measure the response of living mosquitoes to the toxicity of insecticide on sprayed surfaces. The mortality rate of exposed mosquitoes serves as a proxy to indicate how well an SOP applied insecticide on the walls of a house.^27^ Each district used the same insecticide from the carbamate class (bendiocarb).

In 2013, the study team selected 1 village from each of the 6 CB IRS districts (including the pilot district Kersa) and 1 village each from 2 of the matched DB IRS districts to assess the quality of spraying. With a limited number of mosquitoes available to perform the quality checks, the emphasis in 2013 was to ensure that the quality of IRS in the CB IRS model was good; thus, all CB IRS districts were assessed. In 2014, the study team continued the quality check tests in the same 2 districts from the DB IRS model and in 2 of the 6 districts from the CB IRS model.

For the CB IRS model, in 2013 the study team used a 2-stage random sampling method to select the villages and then the houses in each village to conduct the quality control tests using wall bioassays. In 2014, the study team purposively selected 2 districts from the CB IRS model that were adjacent to the 2 districts from the DB model, and then applied the same 2-stage random sampling method to select the villages and then the houses in each village.

For the DB IRS model, the study team used a multi-stage random sampling method to select the districts, then the villages, and then the houses in each village. In the second stage, the study team randomly picked 1 village from the list of all sprayed villages in the district. In the third stage, the team randomly selected 10 houses per village in 2013 and 12 houses per village in 2014. In 2014, the sampling of 12 houses enabled the study team to select 2 houses from each of the 3 common types of wall surfaces (dung, mud, and painted) for separate tests using either susceptible or wild mosquitoes in the selected districts. In all cases, the team conducted wall bioassays 1–7 days after the spraying, using a laboratory-raised colony and wild mosquitoes. The team conducted the wall bioassays as described in the WHO test procedure for bio-efficacy and persistence of insecticides on treated surfaces.^27^

The outcome variable was the number of dead mosquitoes post-exposure to the sprayed wall in the CB and DB IRS districts. Where appropriate, OpenEpi 2 x 2 tables were used for test of significance in mortality differences between the sprayed houses in CB and DB IRS sites.^28^

### Compliance Assessment

The team developed a paper-based checklist that analyzed 13 key questions to compare the compliance with environmental health and safety standards between the 2 IRS modalities. External supervisors collected the data during the 2 years of spray campaigns. Six of the PMI AIRS Ethiopia permanent staff served as external observers and conducted supervisory visits. The sampling for this assessment included 1 operational site from each of the 30 DB IRS districts and 5 operational sites (villages) from each of the 6 CB IRS districts (again, including the pilot district Kersa). In total, the external supervisors collected compliance data from 60 operational sites. Where appropriate, OpenEpi 2 × 2 tables were used for test of significance in compliance differences.^28^ The technical quality of the spray operation and adherence to environmental compliance measures were ensured through intensive supervision by the district, zonal, and regional health offices as well as by PMI AIRS Project staff. Both CB and DB IRS districts followed the same standard operating procedures.

### Cost Assessment

The objective of the cost assessment was to compare CB IRS with DB IRS in terms of overall costs, coverage, cost per structure sprayed, and cost per person protected.

The cost assessment combines (1) a before-after analysis of districts transitioned to CB IRS (i.e., costs in 2012 before CB IRS was implemented compared with costs in 2013 and 2014 after CB IRS was implemented), and (2) a comparison of costs in CB IRS districts with the matched DB IRS districts in 2013 and 2014. The team did not include the initial CB IRS pilot district of Kersa in these analyses because pre-CB IRS data from 2011 were not available. Cost data were collected from the financial systems of the PMI AIRS Project. To the extent possible, quantities (e.g., number of SOPs and number of days each SOP was paid a per diem) were separated from the costs (e.g., the amount of the per diem). The team completed separate cost templates for the years 2013 and 2014 for the DB IRS comparison districts, and for 2012 through 2014 for the CB IRS districts. The team first collected data in 2013 for retrospective costs in 2012 (if applicable) and for costs in 2013. A second round of data collection occurred at the end of 2014. The team converted costs for capital items into annual equivalent costs. The team did not include costs for insecticides in these analyses because they vary directly with the number of structures sprayed. All costs are in 2014 US dollars. The team conducted cost-driver analyses, separating recurrent and capital costs, and assessed the difference in costs associated with inputs that changed with the switch from DB IRS to CB IRS. We used *t* test to determine statistical significance. The study team extracted the coverage data from the project database that tracks all key IRS indicators on an annual basis. For details on the costing methodology and cost categories, see Supplementary Materials 2 and 3.

## RESULTS

### Spray Coverage

In the 5 districts that transitioned to CB IRS in 2013, the average number of eligible structures found increased by 19.6% between 2012 (before CB IRS) and 2013 (during CB IRS), from 19,085 structures to 22,843 structures (*P*=.02) (Table 2). The number of eligible structures sprayed increased by 20.3%, from 18,958 structures to 22,809 structures (*P*=.02). Meanwhile, there was an 8.1% increase in eligible structures found in the DB IRS districts, from 18,797 structures to 20,322 structures (*P*=.11 for the comparison between DB IRS and CB IRS). The number of people protected increased by 8.5% in the CB IRS districts between 2012 and 2013, from about 55,000 people to about 60,000 (*P*=.055).

**TABLE 2.** IRS Coverage by Delivery Model, Selected Districts of Ethiopia, 2012–2014

Coverage Measure	Average per District			Difference (2013–2012)	Difference (2014–2013)	Difference (2014–2012)
	2012	2013	2014			
**CB IRS districts**^a^ **(N=5)**	**DB IRS**	**CB IRS**				
Number of eligible structures found by SOPs	19,085	22,843	26,568	3,758* (19.6%)	3,725 (14.0%)	7,483** (39.2%)
Number of eligible structures sprayed	18,958	22,809	26,365	3,851* (20.3%)	3,556 (13.5%)	7,407** (39.1%)
Spray coverage rate	99.30%	99.90%	99.20%	0.60%	−0.70%	−0.10%
Total population protected	54,902	59,551	60,765	4,649 (8.5%)	1,214 (2.0%)	5,863 (10.7%)
**DB IRS districts (N=5)**	**DB IRS**					
Number of eligible structures found by SOPs	18,797	20,322	20,396	1,525 (8.1%)	74^§§§^ (0.4%)	1,599^++^ (8.5%)
Number of eligible structures sprayed	N/A	20,245	20,347	N/A	102^§§^ (0.5%)	N/A
Spray coverage rate	99.60%	99.80%	0.20%	N/A		
Total population protected	51,871	50,326	−1,545 (−3.0%)	N/A		

Abbreviations: CB IRS, community-based indoor residual spraying; DB IRS, district-based indoor residual spraying; IRS, indoor residual spraying; SOP, spray operator.

a The 5 CB IRS districts transitioned from DB IRS in 2013; 2012 numbers refer to DB IRS coverage before CB IRS was implemented.

* *P*<.05 comparing 2013 with 2012; **P<.01 comparing 2014 with 2012.

++ *P*<.01 for difference in change between DB IRS and CB IRS comparing 2014 with 2012.

§§ *P*<.01 for difference in change between DB IRS and CB IRS comparing 2014 with 2013.

§§§ *P*<.001 for difference in change between DB IRS and CB IRS comparing 2014 with 2013.

The average number of eligible structures found by the spray teams increased more in the community-based IRS districts than the district-based ones.

Between 2013 and 2014, the number of structures found and the number of structures sprayed in the CB IRS districts increased again by a similar order of magnitude (Table 2). The number of people protected increased by 2%, on average. In the 5 comparison DB IRS districts, between 2013 and 2014 the average number of structures found increased by 0.4% (*P*<.001 for comparison between DB IRS and CB IRS), and the number of structures sprayed increased by 0.5% (*P*=.002 for comparison between DB IRS and CB IRS). The number of people protected in the DB IRS districts decreased by 3% between 2013 and 2014 (*P*=.09 for comparison between DB IRS and CB IRS).

### Quality of Spray Operation

In the 2013 spray quality assessment, the mortality of susceptible and wild mosquitoes exposed to sprayed walls 1–7 days after spraying was 99.5% (597/600) in DB IRS districts and 99.9% (1860/1862) in CB IRS districts. There was no significant difference in results between CB and DB IRS (*P*=.18). These results demonstrate comparably good IRS quality in both implementation models.

In 2014, mortality of mosquitoes exposed to sprayed surfaces was 100% on dung and painted surfaces in both the CB and DB IRS districts. There was no difference between CB IRS and DB IRS model sites for the mortality rate of mosquitoes exposed to sprayed houses (96.3% for CB IRS and 95.9% for DB IRS; *P*=.62) (Table 3).

**TABLE 3.** IRS Quality Control Test Results by Delivery Model and Type of Wall Surface, Selected Districts of Ethiopia, 2014

IRS Model	Percent Mortality of Susceptible and Wild Mosquitoes			
	Dung (n=2 houses; 180 mosquitoes)	Mud (n=2 houses; 420 mosquitoes)	Painted (n=2 houses; 300 mosquitoes)	Total (N=6 houses; 900 mosquitoes)
CB IRS (2 districts)	100%	93.6%	100%	96.3%
DB IRS (2 districts)	100%	92.4%	100%	95.9%

Abbreviations: CB IRS, community-based indoor residual spraying; DB IRS, district-based indoor residual spraying; IRS, indoor residual spraying.

### Compliance With Standard Procedures

As shown in Table 4, in 2013, compliance with standard procedures in the CB IRS districts was lower compared with the DB IRS districts (80.8% vs. 91.6%, respectively), and the difference was statistically significant (Yates corrected chi-square=18; *P*<.001). At the time of supervisory visits in 2014, the compliance rate in the CB IRS districts (98.5%) was more or less similar to the compliance rate in the DB IRS districts (100%). The difference between the 2 was not statistically significant (Yates corrected chi-square=3.1; *P*=.07). The following compliance issues were identified: (1) in 1 of 30 supervisory visits to CB IRS districts in 2014, an observer noted an issue with insufficient understanding of procedures for updating stock cards and insecticide tracking forms, (2) in 4 other visits, observers noted an issue with the provision of sufficient washing facilities/showers for the spray operators.

**TABLE 4.** Compliance With AIRS Project IRS Environmental Health and Safety Standards,^a^ by IRS Delivery Model, Selected Districts of Ethiopia,^b^ 2013 and 2014

Year	Overall	CB IRS Sites	DB IRS Sites	Difference in Performance (DB IRS – CB IRS)
2013	84.1%	80.8%	91.6%	10.8 percentage points***
2014	99.2%	98.5%	100.0%	1.5 percentage point

Abbreviations: AIRS, Africa Indoor Residual Spraying; CB IRS, community-based indoor residual spraying; DB IRS; district-based indoor residual spraying; IRS, indoor residual spraying.

a Average compliance scores on a 13-item checklist.

b Data are from 6 CB IRS districts and 30 DB IRS districts. Five operational sites (villages) from each of the 6 CB IRS districts (30 operational sites total) and 1 operational site from each of the 30 DB IRS districts (30 operational sites total) were selected for the compliance assessment.

*** *P*<.001.

### Cost and Efficiency

#### Before-and-After Transition from DB IRS to CB IRS in CB IRS Districts

Total amortized costs increased, on average, by 11.5% per district when the districts transitioned from DB IRS to CB IRS between 2012 and 2013 (*P*=.36) (Table 5). However, increased coverage more than offset the increased cost. Thus, the cost per structure sprayed decreased 9.8% (*P*=.31), and the cost per person protected decreased 1.3% (*P*=.91). Cost per district remained relatively constant in CB IRS districts between 2013 and 2014, increasing 0.6%, while cost per structure sprayed and cost per person protected continued to fall (none of results were statistically significant).

**TABLE 5.** Cost of IRS in 2014 US Dollars, by Delivery Model, Selected Districts of Ethiopia, 2012–2014

Coverage Measure	Average per District			Difference (2013–2012)	Difference (2014–2013)
	2012	2013	2014		
**CB IRS districts (N=5)**^a^	**DB IRS**	**CB IRS**			
Total amortized costs	47,163	52,609	52,930	5,446 (11.5%)	321 (0.6%)
Cost per structure sprayed	2.52	2.27	1.98	−0.25 (−9.8%)	−0.29 (−13.0%)
Cost per person protected	0.88	0.87	0.86	−0.01 (−1.3%)	−0.01 (−1.0%)
**DB IRS districts (N=5)**	**DB IRS**				
Total amortized costs	N/A	48,990	49,665	N/A	675 (1.3%)
Cost per structure sprayed		2.47	2.47		0.00 (0.0%)
Cost per person protected		1	1.03		0.04 (3.3%)

Abbreviations: CB IRS, community-based indoor residual spraying; DB IRS, district-based indoor residual spraying; IRS, indoor residual spraying.

a The 5 CB IRS districts transitioned from DB IRS in 2013; 2012 costs refer to DB IRS coverage before CB IRS was implemented.

Total costs increased on average when districts transitioned from the district- to community-based IRS model but increased coverage under the community-based model offset the increased cost.

The reduction in cost per person protected and per structure sprayed was due to the continued increases in coverage, suggesting greater efficiency. In the DB IRS districts, the costs changed 3% or less between 2013 and 2014 for all 3 indicators (none was statistically significant), similar to the coverage indicators.

In 3 CB IRS districts, costs decreased between 2013 and 2014 compared with 2012 (when the districts were still using the DB IRS model), while in the 2 other districts, the cost per structure sprayed increased compared with 2012. The 2 districts that had increased cost per structure sprayed had the highest absolute increase in both capital and recurrent costs. Analysis of the quantities of inputs employed showed that these 2 districts had the highest increase in the number of SOPs deployed in association with CB IRS. For a cost breakdown per district and per year for all study districts, see Supplementary Material 4.

### Cost and Efficiency

#### CB IRS Districts Compared With Matched DB IRS Districts

**FIGURE 2.**
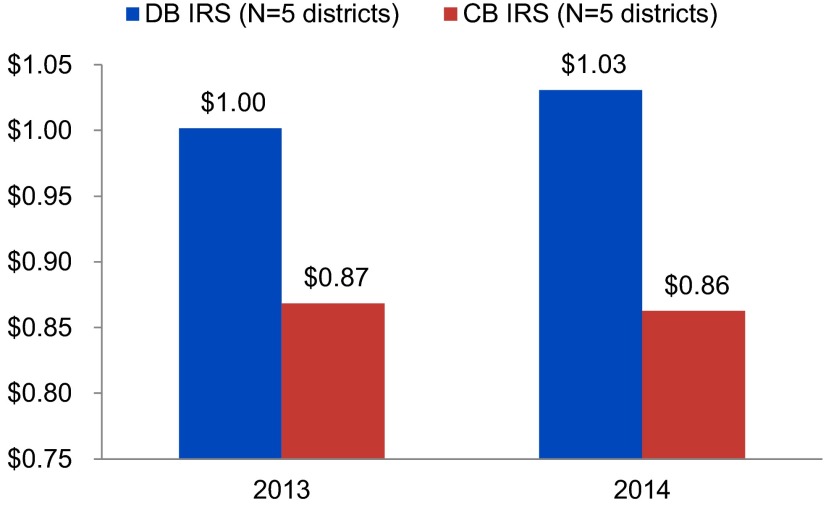
Average Amortized Cost of IRS per Person Protected by Delivery Model, Selected Districts of Ethiopia, 2013 and 2014 Abbreviations: CB IRS, community-based indoor residual spraying; DB IRS, district-based indoor residual spraying; IRS, indoor residual spraying.

The CB IRS districts had higher amortized costs by about US$3,000 per district compared with their matched DB counterparts (e.g., in 2013 US$52,609 vs. US$48,990, respectively) (Table 5). However, the CB IRS districts were better at finding structures and thus sprayed more structures than the DB IRS districts. In 2013, CB IRS districts' average cost per person protected was US$0.13 lower than in DB IRS districts (US$0.87 vs. US$1.00, respectively; *P*=.34), and the difference rose to US$0.16 in 2014 (US$0.86 vs. US$1.03, respectively; *P*=.15) (Figure 2).

## DISCUSSION

### Quality and Compliance

This study shows that the quality of CB IRS operations is good and comparable with the DB IRS model. It suggests that training quality in CB IRS districts was as good as in DB IRS districts. In fact, supervisors noted that in CB IRS, SOPs had more time for interaction with trainers. While the trainings under DB IRS were conducted centrally at the district level with tens, or at times hundreds, of SOPs at one place, under CB IRS the SOP training was done at the village level for only 5 SOPs at a time. The closer interaction between the SOPs and their HEW trainers in the CB IRS model is expected to result in higher-quality training. Another observation was that fewer SOPs used the same washing area compared with the crowded washing areas used in the DB model, which may have been a contributing factor in compliance with performance standards related to the end-of-day cleanup procedures. Rinsing IRS equipment and personal protective equipment is a standardized procedure, requiring a certain number of barrels, that is closely supervised. The CB IRS model, with fewer SOPs per washing area, allows for better clean-up as well as closer supervision, and thus compliance with standards, compared with the DB IRS washing area, which has a substantially higher number of SOPs.

**Figure:**
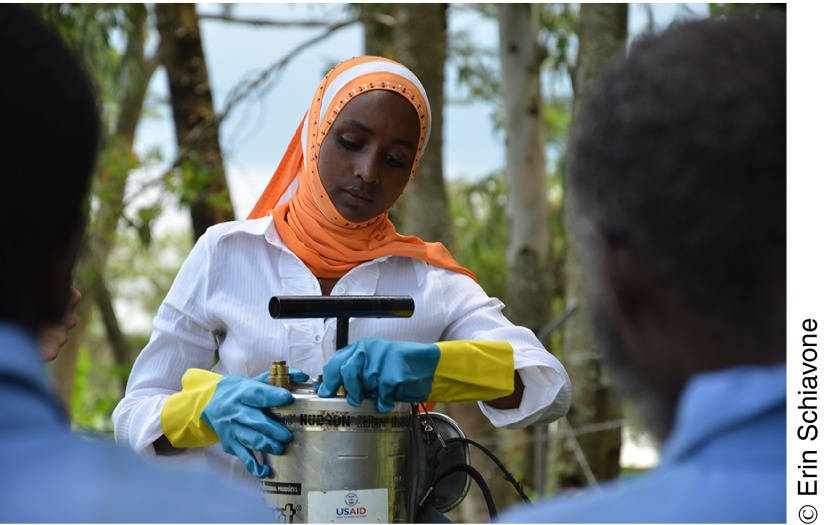
A health extension worker, after receiving training at the district level, returns to her village to train the selected spray operators on indoor residual spraying techniques.

The study team used wall bioassay to assess the quality of the spray operation. Underdosing can be detected using the wall bioassays method and is sufficient to determine if any overdosing occurred. There are currently no effective or cost-efficient methods that can measure the amount of insecticide deposited on a wall surface. Research institutions are working on developing tools to measure insecticide quantification on sprayed surfaces. The PMI AIRS Project does have a routine monitoring tool to check that SOPs are not using excessive insecticides (overdosing). Each day the number of sachets of insecticides used by each SOP is compared against the number of structures sprayed on the same day. If the average number of sachets used exceeds the expectation, the work of the SOP is closely supervised the next day and corrective measures are taken as needed. No such instances occurred during this study.

Although there were some compliance issues during the first year of the CB IRS implementation, these were corrected during 2014 and compliance increased to the DB IRS model level. Compliance improved in the second year of implementation due to corrective measures taken on location during supervision and retraining of the HEWs the following year. HEWs receive 6 days of refresher training each year before the start of the spray operations.

### Coverage

CB IRS appears to result in higher structure and population coverage than DB IRS. In a situation such as that in Ethiopia, where part of a district and even part of the village can be malaria-free and not targeted for IRS, the CB IRS model employs HEWs who use their local knowledge of the demarcations of malaria-affected and malaria-free parts of villages to target spray areas more effectively than in the DB model. This most likely contributed to the increased number of found and sprayed structures under the CB IRS model in both 2013 and 2014. While the study included a relatively small number of districts, the pre-post comparison data from 2012 and 2013/2014 represent a strong counterfactual of the costs of DB IRS since there have been few changes to the DB IRS implementation model since 2012. The counterfactual for the effectiveness of DB IRS is less certain, but the results from the matched comparison indicate that it is unlikely that DB IRS would have had the same increases in coverage as experienced in the CB IRS districts. The results from the second year of CB IRS implementation suggest that the gains in coverage found in the first year of CB IRS will continue in following years.

The findings suggest overall that CB IRS is, under the right conditions, one possible means of increasing the efficiency of malaria control programs.

The community-based IRS model appears to result in higher structure and population coverage than the district-based model.

### Costs

While there were no statistically significant differences in costs between DB IRS and CB IRS, this appears to be due to the fact that costs associated with CB IRS increased in some districts and decreased in other districts. CB IRS appears to result in lower transportation and mobilization costs than DB IRS, but higher costs for training and IRS equipment and supplies. The quantities of inputs employed differ between CB IRS and DB IRS. CB IRS employs more SOPs (mainly reflective of the number of villages in a district), which increases the cost of training, IRS equipment, and supplies. On the other hand, DB IRS has higher transportation costs, reflected in the number of days of rented transport. An increase in the number of SOPs escalates the costs of CB IRS compared with DB IRS, while more days of rented transport under DB IRS indicate more potential savings for CB IRS compared with DB IRS.

The community-based IRS model employs more spray operators than the district-based model, increasing the cost of training, equipment, and supplies, but the district-based model has higher transportation costs.

The cost analysis suggests that in some settings, CB IRS results in lower total costs and greater coverage. Namely, districts that incur relatively high transportation costs under the DB IRS model and/or that require hiring fewer than 35 additional SOPs to implement CB IRS compared with DB IRS likely will have lower total costs with CB IRS. However, in districts with higher increases in SOP numbers, CB IRS might be more costly overall than DB IRS.

The current analysis suggests that districts that require fewer than 40 additional SOPs for CB IRS than for DB IRS are strong candidates for CB IRS. However, when assessed as a relative or percentage increase in the number of SOPs employed, no clear categorization emerged since districts with the largest increase in SOPs under CB IRS also had the most SOPs under DB IRS. Thus, if CB IRS is expanded in Ethiopia or elsewhere, we suggest conducting a needs assessment based on programmatic realities and detailed analysis of what level of staffing is needed under each method.

#### Cost Reduction Opportunities

Modifying how SOPs are deployed in the CB IRS model might reduce costs of the model. The main cost drivers of CB IRS are training, supplies, and equipment to ensure each SOP is well equipped and trained. Currently, more of these inputs are needed in the CB IRS model than in the DB IRS model. While designing the CB IRS model, the study team kept the IRS organizational structure the same as in the DB IRS model: every squad consisted of 4 SOPs in both models. The “community-based” aspect of the design was that 1 squad of 4 SOPs sprayed 1 village irrespective of the number of unit structures found, unlike under the DB IRS model, where a squad sprays more than 1 village over the period of spray operations. As a result, data from 2014 showed that the average number of spray days was 30.5 for DB IRS but only 19.7 days for CB IRS (range, 8 to 34 days). In the DB IRS model, the spray campaign is often completed in about 30 working days uniformly across all districts. Thus, although a larger number of SOPs were trained and provided with the required equipment in the CB IRS districts than the DB IRS districts, the SOPs in many of the CB IRS villages were deployed for a shorter time than their counterparts in the DB IRS model.

If the Government of Ethiopia were to expand the CB IRS model to new districts, we suggest further discussions and analysis on the feasibility of hiring the same number of SOPs as in the DB IRS model and extending the operational time in all areas to around 30 working days. Small villages may use only 1–3 SOPs per squad to finish operations in 30 working days. To spray larger villages, squads may consist of up to 6–7 SOPs. However, of the 6 CB IRS districts in this study, none would have required more than 5 SOPs.

Deploying fewer operators in smaller villages and more operators with increasing village size might reduce costs of the community-based IRS model.

## CONCLUSION

The quality of the spray operation with the community-based IRS model was comparable with the long-established modality of organizing the campaign at the district level. The new community-based model adequately met environmental compliance and safety requirements. The results of the cost analyses suggest that, due to capital costs associated with SOPs, the CB IRS model had, on average, higher total costs but lower unit costs per structure sprayed and person protected than the DB IRS model. Further efforts to rationalize the CB IRS model may reduce the total cost of the intervention and increase its financial sustainability.

During the post-spray review meetings, stakeholders said that communities were more satisfied with CB IRS, and that the quality of training and operation is possibly better than in DB IRS. These findings suggest that CB IRS could be more sustainable and efficient than DB IRS, although further experimentation and testing is needed. The CB IRS model benefited from a preexisting community-based HEP. Moreover, Ethiopia has a history of IRS implementation, and malaria prevention is a routine HEW responsibility. Organization and implementation of IRS through the HEP could enhance efficiency and sustainability of the Ethiopian malaria control and elimination program. However, additional research may be needed to assess whether involvement in IRS impacts other activities of the HEW.

This study provides important lessons for countries that have HEP-like systems and government-supported IRS programs, namely that the government system allows leveraging existing human resources at low or no cost and that these resources (the HEWs) are well trained and educated, which contributes to more efficient performance under the CB IRS model. Countries without an institutionalized community health service system will need to factor in costs and time to establish a function similar to the HEWs to ensure smooth and timely performance of IRS or other public health campaigns. Established community-based service delivery programs can adapt to include a seasonal IRS campaign as part of their routine health prevention activities. Additional research may be appropriate on strategies for further cost reduction and for increasing community contributions. A beneficiary survey comparing the 2 models could also provide insights into the community perception of each model.

## Supplementary Material

Supplementary Material 1

## Notes

**Peer Reviewed**
