# Supplementary material for: Indoor Residual Spraying Delivery Models to Prevent Malaria: Comparison of Community- and District-Based Approaches in Ethiopia
Source: Glob Health Sci Pract. 2016 Dec 23;4(4):529–41. doi: 10.9745/GHSP-D-16-00165 (PMC5199172; doi:10.9745/GHSP-D-16-00165)
Supplement: Supplementary Material 1 [file GHSP-D-16-00165_index.html]

Supplement to Indoor Residual Spraying Delivery Models to Prevent Malaria: Comparison of Community- and District-Based Approaches in Ethiopia | Global Health: Science and Practice

## Supplemental material

- Text s01, PDF - Text s01, PDF
