# Supplementary material for: Indoor Residual Spraying Delivery Models to Prevent Malaria: Comparison of Community- and District-Based Approaches in Ethiopia
Source: Glob Health Sci Pract. 2016 Dec 23;4(4):529–41. doi: 10.9745/GHSP-D-16-00165 (PMC5199172; doi:10.9745/GHSP-D-16-00165)

Johns B, Yihdego YY, Kolyada L, Dengela D, Chibsa S, Dissanayake G, et al. Indoor residual spraying delivery models to prevent malaria: comparison of community- and district-based approaches in Ethiopia. Glob Health Sci Pract. 2016. http://dx.doi.org/10.9745-GHSP-D-16-00165

Supplementary Materials

Supplementary Material 1. Districts Included in the Analyses, Ethiopia, 2012–2014

District	Structures found in 2012	Year 2012	Year 2013	Year 2014	
Sasiga	14,024				Matched
Wayu Tuka	14,074	N/A			
Manasibu	24,373				Matched
Tiro Afeta	23,363	N/A			
Bako Tibe	14,317				Matched
Dano	13,941	N/A			
Hawa Galan	21,162				Matched
Sekoru	20,666	N/A			
Chewaka	22,237				Matched
Borecha	21,940	N/A			

Legend:

	District based
	Community based
N/A	Data not available

Johns B, Yihdego YY, Kolyada L, Dengela D, Chibsa S, Dissanayake G, et al. Indoor residual spraying delivery models to prevent malaria: comparison of community- and district-based approaches in Ethiopia. Glob Health Sci Pract. 2016. http://dx.doi.org/10.9745-GHSP-D-16-00165

Supplementary Material 2. Costing Methodology Details

Analysis Component	Method Employed
Perspective	Program funder – costs incurred by the funder of the program (PMI) were included. Costs to other sectors, e.g., to households, were not included.
Valuation	Financial – all incurred expenditures were included (except certain categories; see assumptions). Other costs, such as opportunity costs for volunteer time, were not included.
Approach	Bottom-up costing. Both quantities and prices were collected whenever possible.
Data collection	Study team developed data collection templates based on program activities. Financial staff at the PMI AIRS office in Addis Ababa filled in data on the cost of each activity. Data were first collected in 2013 for costs in 2012 and 2013; a second round of data collection occurred at the end of 2014. Budgetary line items were first sorted into broad categories.
Unit of analysis	Cost data were collected separately for each district. Total costs of implementation were calculated for each district for each year. Total costs for each district were divided by the number of structures sprayed and the number of people protected in the respective district to calculate unit costs.
Inflation	Prices collected in 2014 were used to calculate costs in 2013 and 2012 whenever possible. If prices were not available for a given year for an item in a particular district, (i) the average change in price over the appropriate years in other districts for that item was applied to the district without prices for a given year, or (ii) if no data were available from other districts, we used the GDP inflation rate[1] to adjust prices. All costs are presented in 2014 US dollars.
Capital items	Annual equivalent costs were calculated using straight-line depreciation. Useful life of capital items were based either on observed replacement rates (for items with useful lives under 4 years such as coveralls, boots, masks, and helmets) or estimated based on interviews with staff (for spray pumps, soak pits, and tents).
Exchange rate	All prices were converted to US dollars at a rate of 19.8 Ethiopian Birr per dollar, which was the average exchange rate for the project at the time of IRS implementation.
Major assumptions	1. Costs for insecticides are not included in these analyses. Any variance from the direct relationship between the number of structures sprayed and the costs of insecticide is due either to variation in the size of structures or the efficiency in the use of insecticides. Including insecticide costs in the comparison may introduce spurious factors since variation in the size of structures between districts is arbitrary. However, by excluding the costs of insecticide, we assume that each implementation model uses insecticide with the same efficiency. Efficiency in the use of insecticide depends mostly on the size of the villages sprayed and the type of spray equipment used, which were the same in both models.<br>2. Overhead costs (e.g., AIRS staff costs in Addis Ababa operating the program) were not included in the analyses because we assume that these costs do not change

[1] International Monetary Fund. October 2014. World Economic Outlook Database. http://www.imf.org/external/pubs/ft/weo/2014/02/weodata/weorept.aspx?sy=2012&ey=2014&scsm=1&ssd=1&sort=country&ds=.&br=1&c=644&s=NGDP_D%2CNGDPPC%2CNGDPDPC&grp=0&a=&pr1.x=31&pr1.y=1. Accessed December 13, 2014.

	markedly in association with the switch from DB IRS to CB IRS (since, in general, districts receive similar levels of support from the Addis Ababa office).

Abbreviations: AIRS, Africa Indoor Residual Spraying; CB IRS, community-based indoor residual spraying; DB IRS, district-based indoor residual spraying; GDP, gross domestic product; IRS, indoor residual spraying; PMI, United States President's Malaria Initiative.

Johns B, Yihdego YY, Kolyada L, Dengela D, Chibsa S, Dissanayake G, et al. Indoor residual spraying delivery models to prevent malaria: comparison of community- and district-based approaches in Ethiopia. Glob Health Sci Pract. 2016. http://dx.doi.org/10.9745-GHSP-D-16-00165

Supplementary Material 3. IRS Implementation Cost Categories and Expenditure Items: DB IRS vs. CB IRS in Selected Districts of Ethiopia, 2012–2014

Cost Category	DB IRS Implementation	CB IRS Implementation
IRS Supplies	Boots, Coveralls, Helmets (with face shields), Masks, Pumps, **Camping Tents**	Boots, Coveralls, Helmets (with face shields), Masks, Pumps
Operations	Per diems for:<br>• **District Supervisor**<br>• **Squad Team Leader**<br>• SOPs<br>• Data clerks<br>• Guard/ Washer<br>Water for washing<br>Loading/unloading IRS equipment<br>Soak pit construction/renovation	Per diems for:<br>• **Supervisor**<br>• **HEWs**<br>• SOPs<br>• Data clerks<br>• Guard/Washer<br>Water for washing<br>Loading/unloading IRS equipment<br>Soak pit construction/renovation
Training	Per diems for:<br>• Mobilizers<br>• Trainers of data clerks<br>• **Squad Leader trainees**<br>• **Squad Leader trainers and SOP trainees**	Per diems for:<br>• Mobilizers<br>• Trainers of data clerks<br>• **District staff trainers and HEW trainees**<br>• **HEW trainers and SOP trainees**
Transportation	Car rental (fuel costs included)	**Fuel for motorbikes** (machines provided by government), car rental
Other	Communication costs, SOP forms, per diems for Mobilizers, and miscellaneous IRS equipment expenses	Communication costs, SOP forms, per diems for Mobilizers, and miscellaneous IRS equipment expenses

Note: Differences in line items between the two implementation methods are highlighted in boldface.

Abbreviations: CB IRS, community-based indoor residual spraying; DB IRS, district-based indoor residual spraying; HEW, health extension worker; IRS, indoor residual spraying; SOP, spray operator.

Johns B, Yihdego YY, Kolyada L, Dengela D, Chibsa S, Dissanayake G, et al. Indoor residual spraying delivery models to prevent malaria: comparison of community- and district-based approaches in Ethiopia. Glob Health Sci Pract. 2016. http://dx.doi.org/10.9745-GHSP-D-16-00165

Supplementary Material 4. Cost Calculations for the CB IRS and DB IRS Models, per District per Year, Ethiopia

SUPPLEMENTARY TABLE 4A. TOTAL COST FOR DISTRICTS ADOPTING CB IRS IN 2013, PER DISTRICT PER YEAR

District	Year	IRS Supplies	Operations	Other	Training	Transportation
Chewaka	2012	$6,015	$18,979	$2,980	$4,189	$12,935
Chewaka	2013	$15,657	$34,478	$400	$12,680	$4,507
Chewaka	2014	$17,127	$32,270	$121	$13,327	$5,328
Bako Tibe	2012	$4,920	$21,398	$1,687	$3,274	$14,163
Bako Tibe	2013	$7,597	$22,096	$288	$6,356	$3,999
Bako Tibe	2014	$8,591	$21,148	$81	$6,878	$4,065
Hawa Galan	2012	$9,428	$22,670	$1,989	$5,150	$17,683
Hawa Galan	2013	$12,590	$34,122	$365	$10,017	$4,292
Hawa Galan	2014	$13,468	$29,228	$104	$10,721	$8,290
Manasibu	2012	$8,933	$23,239	$2,532	$4,309	$16,177
Manasibu	2013	$14,334	$35,717	$405	$11,381	$4,316
Manasibu	2014	$14,997	$33,512	$112	$12,382	$4,422
Sasiga	2012	$3,237	$14,990	$891	$2,126	$11,922
Sasiga	2013	$5,209	$13,918	$234	$4,065	$4,023
Sasiga	2014	$5,542	$14,234	$66	$4,570	$4,065
Average	2012	$6,507	$20,255	$2,016	$3,810	$14,576
Average	2013	$11,077	$28,066	$338	$8,900	$4,228
Average	2014	$11,945	$26,078	$97	$9,576	$5,234

Abbreviations: Abbreviations: CB IRS, community-based indoor residual spraying; DB IRS, district-based indoor residual spraying; IRS, indoor residual spraying.

Johns B, Yihdego YY, Kolyada L, Dengela D, Chibsa S, Dissanayake G, et al. Indoor residual spraying delivery models to prevent malaria: comparison of community- and district-based approaches in Ethiopia. Glob Health Sci Pract. 2016. http://dx.doi.org/10.9745-GHSP-D-16-00165

SUPPLEMENTARY FIGURE 4A. TOTAL COST FOR DISTRICTS ADOPTING CB IRS IN 2013, PER DISTRICT PER YEAR

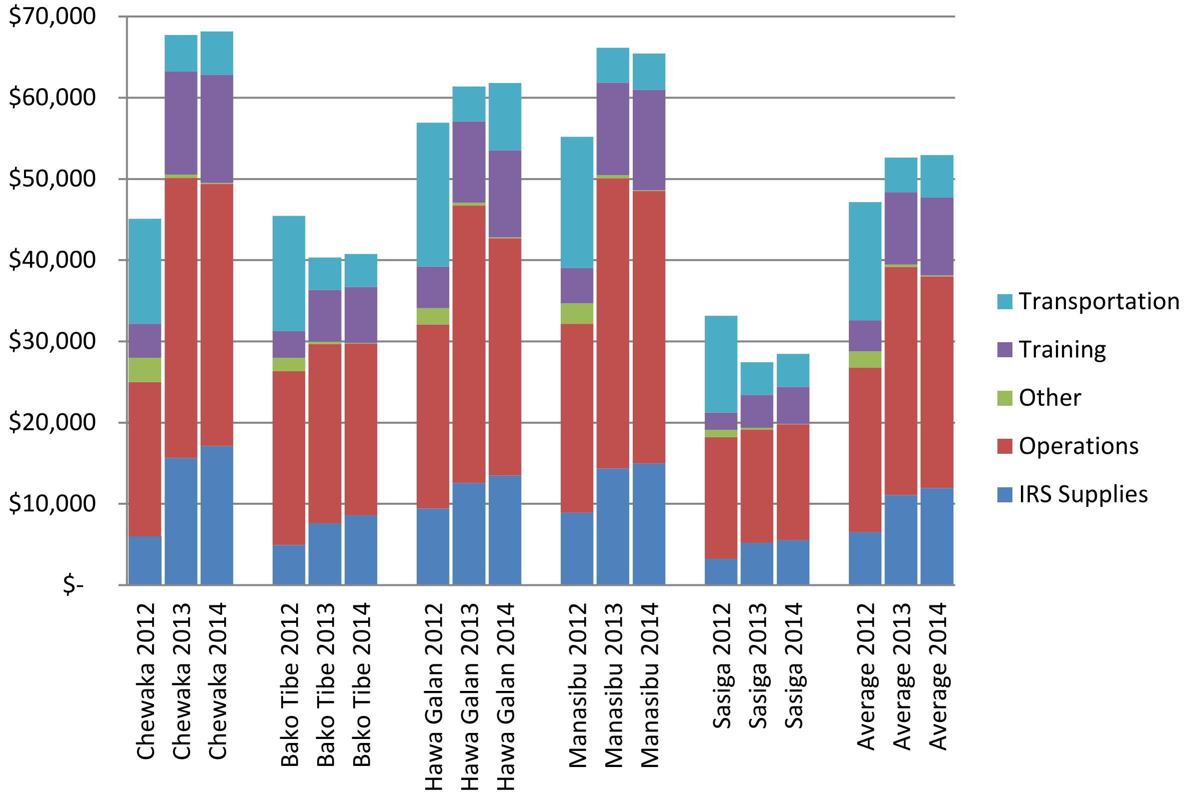

Johns B, Yihdego YY, Kolyada L, Dengela D, Chibsa S, Dissanayake G, et al. Indoor residual spraying delivery models to prevent malaria: comparison of community- and district-based approaches in Ethiopia. Glob Health Sci Pract. 2016. http://dx.doi.org/10.9745-GHSP-D-16-00165

SUPPLEMENTARY TABLE 4B. TOTAL COST FOR DISTRICTS EMPLOYING ONLY DB IRS, PER DISTRICT PER YEAR

District	Year	IRS Supplies	Operations	Other	Training	Transportation
Borecha	2013	$6,372	$22,499	$1,409	$3,419	$11,741
Borecha	2014	$6,572	$22,210	$1,609	$3,371	$8,610
Danno	2013	$5,059	$21,968	$1,132	$2,825	$11,588
Danno	2014	$5,259	$21,579	$1,713	$2,825	$15,450
Sekoru	2013	$7,420	$23,256	$1,633	$3,551	$15,582
Sekoru	2014	$6,572	$20,925	$1,884	$3,551	$15,582
Tiro Afeta	2013	$8,313	$26,985	$1,533	$4,280	$19,477
Tiro Afeta	2014	$8,595	$26,911	$1,950	$4,319	$23,373
Wayu Tuka	2013	$5,534	$19,435	$1,262	$3,020	$15,655
Wayu Tuka	2014	$5,916	$19,607	$1,188	$3,098	$15,655
Average	2013	$6,540	$22,829	$1,394	$3,419	$14,808
Average	2014	$6,583	$22,246	$1,669	$3,433	$15,734

Johns B, Yihdego YY, Kolyada L, Dengela D, Chibsa S, Dissanayake G, et al. Indoor residual spraying delivery models to prevent malaria: comparison of community- and district-based approaches in Ethiopia. Glob Health Sci Pract. 2016. http://dx.doi.org/10.9745-GHSP-D-16-00165

SUPPLEMENTARY FIGURE 4B. TOTAL COST FOR DISTRICTS EMPLOYING ONLY DB IRS, PER DISTRICT PER YEAR

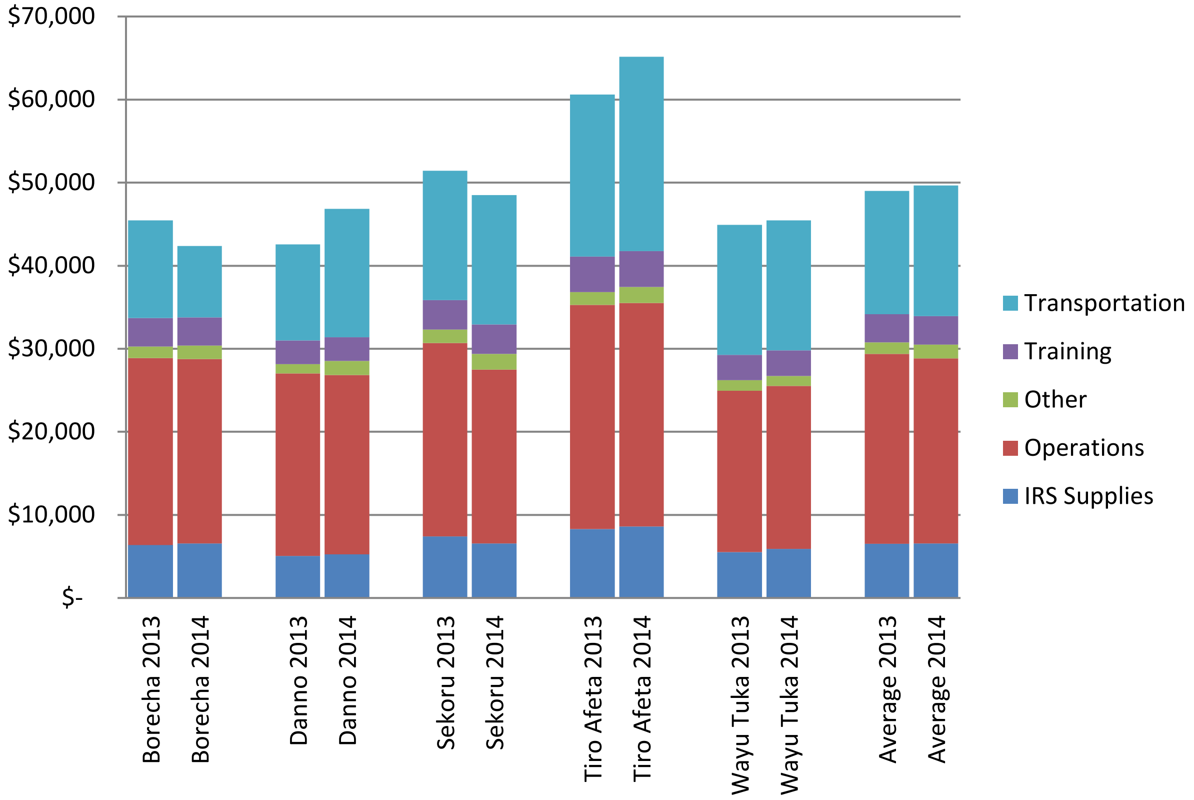

Supplement: Supplementary Material 1 [file 16-00165-Kolyada-Supplementary-Materials_1-4.pdf]
